# Surveying and mapping cereals and legumes wild relatives in Mount Hermon (Bekaa, Lebanon)

**DOI:** 10.1002/ece3.10943

**Published:** 2024-03-11

**Authors:** Eliane Sayde, Lamis Chalak, Safaa Baydoun, Ali Shehadeh, Hicham El Zein, Jostelle Al Beyrouthy, Mariana Yazbek

**Affiliations:** ^1^ Faculty of Agronomy, Department of Plant Production Lebanese University Beirut Lebanon; ^2^ Research Center for Environment and Development Beirut Arab University Bekaa Lebanon; ^3^ International Center for Agricultural Research in Dry Areas (ICARDA) Beirut Lebanon; ^4^ Independent Researchers Beirut Lebanon

**Keywords:** botanical survey, cereals and legumes, CWR, distribution mapping, DIVA‐GIS, mount Hermon

## Abstract

Crop Wild Relatives (CWR) should be highly prioritized, monitored, and conserved as they have an immense effect on sustainability and livelihood. In this study we aim to survey and map cereal and legume wild relatives of Fabaceae and Poaceae families. Mount Hermon, Bekaa side, Lebanon. A set of 46 CWR species were targeted based on desk selection analysis and prioritization by the International Center for Agricultural Research in Dry Areas genebank for their potential importance in breeding programs. A botanical survey of 17 sites of the various habitats of Mount Hermon was performed during April–June 2021 using a systematic transect/quadrate sampling method. Recorded genera and species were accurately georeferenced and then mapped with the DIVA‐GIS program. In total, 854 occurrences were observed belonging to 34 species of Fabaceae and 12 species of Poaceae. High H′ Shannon diversity values were recorded in three sites (Al Fakiaa, Sham El Hafour and Ain Ata‐ al Berke) of the Mount with values ranking between 2.45 and 2.83. This was confirmed by the richness distribution maps of genera and species. Richness distribution maps provide relevant clues on candidate sites for high concentrations of each of the species under study. At least the three sites, hosting 87% of the surveyed CWR's species, can be considered for further in situ conservation actions.

## Graphical abstract

In this study we aim to survey and map the distribution, diversity, and richness of targeted cereal and legume wild relatives of Fabaceae and Poaceae families in Mt. Hermon of Lebanon, Bekaa side. Richness distribution and predictive maps provide relevant clues on candidate sites for conservation planning.

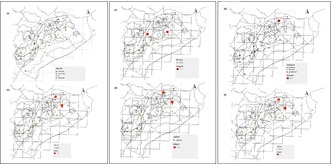

## 1 INTRODUCTION

Crop Wild Relatives (CWR) are a valuable source of useful diversity that can improve crop performance to adapt to ever‐changing conditions and to overcome the constraints caused by pests, diseases, and abiotic stresses (Bohra et al., 2021, Perrino & Wagensommer, 2021). They represent an extended gene pool that can be tapped for the breeding of novel varieties with adaptability traits to various environmental stresses (Maxted et al., 2016). CWR are also essential for sustainable agriculture and to maintain agrobiodiversity in a scenario of climate change and its unpredictable outcomes (Bierschenk et al., 2020). Nevertheless, many CWR genetic resources are threatened in their natural habitats and/or not fairly represented in genebank and conservation initiatives (Bohra et al., 2021; Castañeda‐Álvarez et al., 2016; Ceccarelli et al., 2012; Kell et al., 2012). These conservation gaps limit therefore the range of useful plant diversity that should be available for present and future generations (Khoury et al., 2019). These CWR should be considered as a priority for conservation, monitoring, and sustainable utilization.

Technology‐based approaches such as generating distribution maps are deployed as very important tools to illustrate the current richness of CWR, predicted future richness, and its changing trends (Jarvis et al., 2008). Such maps are developed as a valuable tool for guiding the implementation of appropriate conservation strategies. More specifically, they can be used for prioritizing the hotspot areas for in situ conservation such as the establishment of CWR reserves (Ratnayake et al., 2021). For instance, mapping the richness and distribution of prioritized CWR in Zambia helped identify sites for active in situ conservation as a part of a national plan for the development of conservation and sustainable use of CWR (Ng'uni et al., 2019).

Located at the Eastern shore of the Mediterranean Basin, Lebanon represents one of the most important agrobiodiversity hotspots, not only for wild species diversity but also for cultivated plant diversity (Pacicco et al., 2018). Lebanon is also well recognized for its wealth in CWR (Nail, 2019), with a large share of Poaceae and Fabaceae families (Chalak et al., 2020; Zair et al., 2018). The country is also part of the Fertile Crescent that is known to have been the cradle of agriculture as having a very rich and unique biodiversity of flora owed to its mountainous topography and the great diversity of its climatic conditions (Chalak et al., 2011, 2020). It has a high percentage (12%) of endemic plant species which are mostly located on the high summits of the two mountain ranges spanning parallel to the sea coast (i.e., Lebanon Mountains and Anti‐Lebanon Mountains) (Chalak et al., 2020).

Mount Hermon, also known as Jabal al‐Shaykh, is a mountain cluster constituting the southern end of the Anti‐Lebanon Mountains range (Baydoun et al., 2015). The mountain offers a wide range of ecosystem products mainly in terms of fresh water and agricultural crops. Mount Hermon was described to be a high mountain plateau characterized by the rarity, richness, and uniqueness of vascular plants. It was identified as an Important Plant Area in Lebanon where endemic and/or threatened species are found (Bou Dagher Kharrat et al., 2018; IUCN, 2001) and where more than hundred species specific to Mount Hermon and the Anti‐Lebanon range have been counted (Arnold et al., 2015; Medail & Quezel, 1997). A Richness Index (i.e., a numerical score assigned to each cell based on the number of species present, using a scale ranging from 0 to 5 and calculated using the Jenks natural breaks classification method (1967)) scored 4 referring to 259 species harbored by the Mount was reported (Bou Dagher Kharrat et al., 2018). Many of these species are characteristic of the semiarid Eastern Mediterranean climate and are endemic to Lebanon and Syria (Arnold et al., 2015; Bou Dagher Kharrat et al., 2018). The natural wealth of Mount Hermon has been subject to pressures impacting its plants and all other types of wildlife. Habitat fragmentation, overexploitation of resources, grazing, invasive alien species, urban expansion, and increasing impact of climate change are among the challenges facing the mountain and its surrounding communities (Arnold et al., 2015).

Recently and within its mission of collecting, conserving, and utilizing dryland agrobiodiversity, the International Center for Agricultural Research in Dry Areas (ICARDA) took the initiative to survey and inventory the CWR growing in Lebanon, and more particularly in the Bekaa province to bridge the gaps of ex situ conservation (Amri et al., 2016; Nail, 2019). In this study, we aim to survey and map CWR in the mountain with an emphasis on Fabaceae and Poaceae species particularly selected for their genetic and economic importance. The study will help bridging the CWR gaps of ICARDA genebank for potential use in breeding programs. It will also provide baseline knowledge to identify hotspots for CWR in situ conservation and management.

## 2 MATERIALS AND METHODS

### 2.1 Study area

This study was conducted on the western slopes of Mount Hermon, Bekaa side, covering an area of about 190 km^2^ that extends between 1067 and 2800 m of altitude. The study area was delimited using a tool in DIVAGIS which is set to layer an ecogeographic grid dividing the study area into georeferenced cells (48 cells) of 2 × 2 km (i.e., 4 km^2^ quadrants), covering the entire area. Seventeen cells out of the total 48 cells representing the different habitats described by Chalak et al., n.d. and capturing the heterogeneity of plant communities of the Mount while considering accessibility were selected based on their accessibility on Mount Hermon (Table 1). The location of Mount Hermon study area in Lebanon is visualized in Figure S1.

**TABLE 1:** Surveyed areas in Mount Hermon with their respective latitude, longitude, altitude, and general dominating habitats.

Study sites	Latitude	Longitude	Altitude (m)	Habitat (Chalak et al. (n.d.))
Tanoura‐ Al Jouk	33.46647	35.80224	1104	Woodland—*Quercus coccifera* Rocky grassland
Arid El Dalia	33.469006	35.8216	1120	Woodland—*Quercus coccifera*
Al Fakiaa	33.45338	35.8132	1135	Woodland—*Quercus coccifera*
Sahel Ayha‐Wadi Al Byara	33.50786	35.8668	1173	Agriculture
Wadi El Raheb	33.44057	35.81472	1219	Woodland—*Quercus infectoria*
Sham El Hafour	33.430588	35.80259	1278	Agriculture Woodland—*Quercus infectoria*
Hima El Kadirin	33.447124	35.822078	12 79	Woodland—*Quercus coccifera* Woodland—*Quercus infectoria*
Al Yebse‐ Wadi El Botem	33.47244	35.846858	1317	Woodland and grassland—*Quercus infectoria* Woodland—*Quercus infectoria*
Ain Ata‐ Marj El Tout	33.488559	35.88283	1335	Thickets of *Quercus* and *Crataegus* Agriculture
Ain Ata‐Wadi Ain El Sayegh	33.41904	35.80208	1373	Thickets of *Quercus* and *Crataegus*
Ain Ata‐Jabal El Khan	33.41807	35.78079	1404	Woodland—*Quercus infectoria*
Rashaya El Wadi‐Wadi Samouk	33.472748	35.866075	1409	Thickets of *Quercus* and *Crataegus*
Ain Ata‐ Al Berke	33.432384	35.782907	1409	Agriculture Woodland—*Quercus coccifera*
Rouwayset El Rafiaa‐Sammouk	33.462342	35.8852	1554	Thickets of *Quercus* and *Crataegus*
Chebaa‐Ouyoun Jenaa	33.3909	35.791365	1575	Woodland—*Prunus dulcis–Quercus look*
Jourit Al Nkar–Al Chok	33.448598	35.845698	1637	Thickets of *Quercus* and *Crataegus* Woodland—*Prunus dulcis–Quercus look*
Fahet Jernaya‐ Beb El Hawa	33.425322	35.839478	2254	Oro‐Mediterranean shrublands, rocks and screes

*Note*: Latitude and longitude correspond to the center of the cells.

### 2.2 CWR targeted species

A desk analysis of various published sources including Vincent et al. (2013) global priority list of CWR, [Global Biodiversity Information Facilities (GBIF, 2021) (http://www.gbif.org)], Genesys (https://www.genesys‐pgr.org/), Euro Med Plant Base (http://www. emplantbase.org), the New Flora of Lebanon and Syria (Mouterde 1966, 1970; Mouterde, 1986) and Lebanon‐Flora (http://www. lebanon‐flora.org), and a dissertation (Al‐Atawneh et al., 2008); and literature related to the flora in Mount Hermon (Baydoun et al., 2015) was first conducted as a pre‐selection step of CWRs found in the Mount. Following this step, 15 CWRs genera belonging to Fabaceae and Poaceae which are two major families present in Lebanon were selected for this study based on their genetic and economic values according to their importance in ICARDA's gene bank which is the main ex situ conservation center for cereals, food legumes, forage, and rangeland species in the country, as well as continuously promoting the utilization of these wild species in experiment targeting world food security. Over these 15 CWRs genera, eight represented Fabaceae consisting of *Lathyrus*, *Lens*, *Medicago*, *Pisum*, *Trifolium*, *Trigonella*, *Coronilla*, and *Vicia*, and the remaining seven genera belonged to Poaceae including *Aegilops*, *Avena*, *Bromus*, *Hordeum*, *Lolium*, *Poa*, and *Triticum*.

### 2.3 Botanical survey

Ten field botanical survey missions were conducted in the natural habitats of the mountain between April and June 2021, exploring the 17 sites, assisted by a guide from the local habitants. Transect/quadrates survey method was used to assess the CWR status. We took into consideration the cells that represent the various types of habitats, noting that some cells designate more than one habitat type. Consequently, each designated cell had to include two transects positioned in a manner to adequately represent the plant communities growing in these habitats of Mount Hermon. Each transect was around 100 m trajectory and comprised five quadrates of 1 m^2^ with an interval distance of 25 m. A total of 170 quadrates were surveyed. Data related to the site coordinates, general geomorphology, general habitat, land cover, and slope were collected.

For the CWR species, records of their Richness, Occurrence, Frequency, and Density were collected, with species nomenclature based on Mouterde (1966, 1970, Mouterde, 1986), the online Euro Med Plant Base (2006) (http://www. emplantbase.org), and the help of specialized botanists.

#### 2.3.1 Species indices

Indices related to species considered in this study were: (i) *Species occurrence* considered to be the species presence/absence in the assessed 170 quadrates; (ii) *Species density* defined as the number of individuals of a given species that occurs within a quadrate (MacKenzie et al., 2018); (iii) *Species frequency* calculated as the percentage of occurrences in the 170 quadrates assessed (Good, 1953).

#### 2.3.2 Diversity indices per site

(i) *Genus richness* and *species richness* being a useful measure of taxonomic diversity (Gaston, 1996) and calculated as number of different genera or species per site (representing the 17 cells); (ii) *Shannon diversity index (H′)* that allows to quantify the species diversity per area unit (cell) using the following formula (Magurran, 1998; Rajan, 2001):

$$H^{\prime }=-\sum \left({\mathrm{Pi}}^{*}\;\ln\;\mathrm{Pi}\right).$$

where Pi is the proportion (*n*/*N*) of individuals of one particular species found (*n*) divided by (*N*) total number of individuals found, ln Pi is the natural log of Pi, and ∑ is the sum of Pi calculations. Species with individual count below 50 were registered as densities (i.e., number of individuals of a species within a quadrate considering that quadrates are representative of the whole cell), while species with individual count equal or greater than 50 were taken as 50 for the sake of the calculation.

## 3 CWR MAPPING

Data collected in botanical surveys of targeted CWR were mapped based on calculated indices described above.

### 3.1 Genus and species mapping

DIVA‐GIS version 7.1.7 was used to map each CWR genera and species distribution in Mount Hermon using data entry tables of identified genera and species with their nomenclature and respective accurate georeferenced occurrence locations collected during the botanical surveys, alongside their densities in quadrates.

## 4 RESULTS

### 4.1 Species occurrence, frequency, and density of targeted CWRs

In this study, 859 georeferenced observations in Mount Hermon were registered for 46 CWR species belonging to the 15 targeted genera, with 12 Poaceae species and 34 Fabaceae species. The most represented genus is *Trifolium* with 17 species, followed by *Medicago* with eight species, while genera like *Bromus*, *Poa*, *Coronilla*, and *Pisum* are represented with only one species (Table 2).

**TABLE 2:** List of targeted CWRs in Mount Hermon, with family, frequencies (occurrences), and range of densities in quadrates where they occurred.

Species	Family	Related crop	Species frequency (%) (number of species occurrence in quadrates)	Density range in quadrates (minimum–maximum)
*Ae. biuncialis* Vis.	Poaceae	Wheat	5.29% (9)	1–50
*Ae. geniculata* Roth.	Poaceae	Wheat	4.12% (7)	1–50
*Ae*. sp.	Poaceae	Wheat	51.76% (88)	2–50
*Ae. triuncialis* L.	Poaceae	Wheat	0.59% (1)	0–17
*A. sterilis* L.	Poaceae	Oat	58.24% (99)	1–50
*B. sterilis* L.	Poaceae	Brome grass	19.41% (33)	1–50
*Coronilla scorpioides* L.	Fabaceae	Vetch	5.88% (10)	1–20
*H. bulbosum* L.	Poaceae	Barley	18.82% (32)	1–27
*H. murinum* subsp. *glaucum (Steud.) Tzvelev*	Poaceae	Barley	12.35% (21)	1–50
*H. spontaneum* C. Koch	Poaceae	Barley	20.59% (35)	1–50
*L. aphaca* L.	Fabaceae	Pea	10% (17)	1–50
*L. blepharicarpus* Boiss.	Fabaceae	Pea	7.65% (13)	1–24
*Lens culinaris* subsp. *orientalis* (Boiss.) Ponert	Fabaceae	Lentil	2.94% (5)	2–50
*Lolium rigidum* Goud.	Poaceae	Ryegrass	25.29% (43)	1–50
*M. coronata* (L.) Bart.	Fabaceae	Alfalfa	2.35% (4)	0–1
*M. minima* (L.) Bart.	Fabaceae	Alfalfa	0.59% (1)	0–1
*M. orbicularis* L.	Fabaceae	Alfalfa	2.35% (4)	2–12
*M. praecox* DC.	Fabaceae	Alfalfa	4.71% (8)	1–50
*M. radiata* L.	Fabaceae	Alfalfa	1.18% (2)	3–13
*M. rigidula* var. *rigidula* (L.) Desr.	Fabaceae	Alfalfa	2.35% (4)	1–4
*M. rotata* var. *rotate* Boiss.	Fabaceae	Alfalfa	8.82% (15)	1–17
*M. sativa* L.	Fabaceae	Alfalfa	0.59% (1)	0–50
*Pisum fulvum* Sibth. & Sm.	Fabaceae	Pea	1.18% (2)	4–6
*Poa pratensis* L.	Poaceae	Meadowgrass	2.35% (4)	5–8
*T. arvense* L.	Fabaceae	Clover	1.18% (2)	2–12
*T. boissieri* Guss.	Fabaceae	Clover	18.82% (32)	1–50
*T. bullatum* Boiss. & Hausskn. var. *bullatum*	Fabaceae	Clover	2.94% (5)	2–12
*T. campestre* Schreb.	Fabaceae	Clover	2.94% (5)	2–50
*T. clusii* Gren. & Godr var. *clusii*.	Fabaceae	Clover	1.18% (2)	0–2
*T. fragiferum* L.	Fabaceae	Clover	0.59% (1)	0–50
*T. glanduliferum* Boiss.	Fabaceae	Clover	5.88% (10)	3–50
*T. hirtum* All.	Fabaceae	Clover	1.76% (3)	4–28
*T. pilulare* Boiss.	Fabaceae	Clover	22.94% (39)	1–50
*T. purpureum* Loisel. var. *purpureum*	Fabaceae	Clover	8.82% (15)	1–50
*T. resupinatum* var. *resupinatum* L.	Fabaceae	Clover	54.12% (92)	1–50
*T. scabrum* L.	Fabaceae	Clover	2.94% (5)	1–20
*T. scutatum* Boiss.	Fabaceae	Clover	34.12% (58)	1–50
*T. spumosum* L.	Fabaceae	Clover	0.59% (1)	0–21
*T. stellatum* L.	Fabaceae	Clover	61.18% (104)	1–50
*T. subterraneum* var. *brachycalycinum* Katzn. & Morley	Fabaceae	Clover	0.59% (1)	0–1
*T. tomentosum* var. *glabrescens* (Post) Hausskn. & Bornm.	Fabaceae	Clover	5.29% (9)	1–50
*Trigonella spinosa* L.	Fabaceae	Fenugreek	0.59% (1)	0–7
*Triticum aestivum* L.	Poaceae	Wheat	0.59% (1)	0–1
*Vicia sativa* L.	Fabaceae	Vetch	0.59% (1)	0–18
*Vicia tenuifolia* Roth.	Fabaceae	Vetch	4.71% (8)	1–8

*Trifolium stellatum* L. had the highest frequency in quadrates of 61.18% with 104 occurrences, followed by *Avena sterillis* L. (58.24% frequency and 99 occurrences), *T. resupinatum* var. *resupinatum* (54.12% frequency and 92 occurrences). Oppositely, nine species recorded only one occurrence each, designating 0.59% on the frequency chart, such as *M. minima* (L.) Bart., *M. sativa* L., *T. fragiferum* L., *T. spumosum* L. and *V. sativa* L. The majority of Poaceae species registered high frequency records between 12.35% and 58.24%, with the *Hordeum* species scoring different occurrence frequencies, *H. murinum* subsp. *glaucum* (Steud.) Tzvelev (12.35%), *H. bulbosum* L. (18.82%), and the highest occurrence frequency for this genus was *H. spontaneum* C. Koch (20.59%). As for Fabaceae species, only few recorded a relatively high frequency value mainly for species belonging to the *Trifolium* genus, *T. pilulare* Boiss. (22.94%) and *T. scutatum* Boiss. (34.12%). Species densities per quadrate fluctuated for the same species depending on the habitat types. All the Poaceae species were present in high densities within the quadrates, as most values were in the range of (1–50), except for *Poa* (5–8) and *Triticum* (0–1) genera that had low species density. For Fabaceae, density ranges were highly fluctuating within the same genera such as *T. boissieri* Guss. scores (1–50) and *T. subterraneum* var. *brachycalycinum* Katzn. & Morley which counted only one time in a quadrate.

### 4.2 Diversity indices of targeted CWRs

The surveyed sites varied in their genus and species richness (Table 3). Up to 35 species belonging to 12 genera were found in Sham El Hafour, 36 species belonging to 9 genera in Al Fakiaa, and 24 species belonging to 9 targeted genera in Ain Ata‐ Al Berke. Fahet Jernaya‐ Beb El Hawa scored the lowest richness with 6 species belonging to 4 genera.

**TABLE 3:** Shannon diversity Index for targeted CWRs, the genus richness, and species richness in surveyed areas of Mount Hermon.

Site	Genus richness	Species richness	Shannon index
Al Fakiaa	9	36	2.71
Sham El Hafour	12	35	2.83
Wadi El Raheb	9	21	2.41
Ain Ata‐Jabal El Khan	6	17	2.27
Ain Ata‐Wadi Ain El Sayegh	8	11	1.64
Rashaya El Wadi‐Wadi Samouk	7	18	2.03
Rouwayset El Rafiaa‐Sammouk	7	12	1.998
Jourit Al Nkar–Al Chok	9	15	2.19
Al Yebse‐ Wadi El Botem	7	14	1.67
Hima El Kadirin	11	24	2.27
Arid El Dalia	7	16	1.58
Chebaa‐Ouyoun Jenaa	8	15	1.23
Ain Ata‐ Al Berke	9	24	2.45
Ain Ata‐ Marj El Tout	6	14	1.89
Fahet Jernaya‐ Beb El Hawa	4	6	1.45
Sahel Ayha‐Wadi Al Byara	6	23	1.65
Tanoura‐ Al Jouk	9	15	2.29

Shannon index representing species richness in the assessed cells was calculated to describe and compare the CWR taxonomical diversity among the visited areas of Mount Hermon. The index fell in the range of 1.23 and 2.82 with the majority of the cells demonstrating high important values, particularly in the sites of Sham El Hafour (2.83), Al Fakiaa (2.71), Ain Ata‐ Al Berke (2.45), and Wadi El Raheb (2.41) which were the most diversified in targeted CWR species compared to other sites in the Mount.

### 4.3 CWR mapping

#### 4.3.1 CWR mapping based on genus richness

**FIGURE 1:**
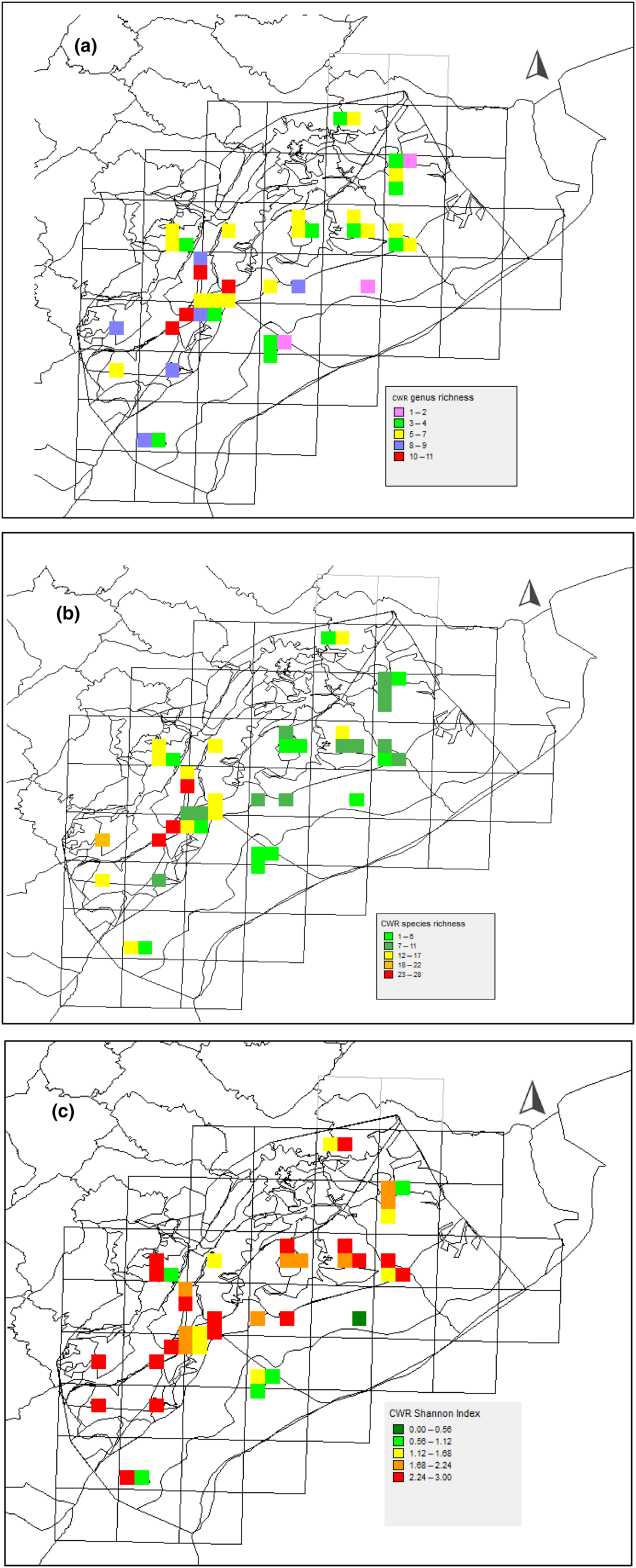
(a) Distribution map of CWR genus richness, (b) CWR species richness, and (c) Map representing CWR diversity ranges using Shannon index in Mount Hermon (generated by DIVA‐GIS).

The sites with the highest CWR genus richness were found in Al‐Fakiaa, Hima El Kadirin, and Sham El Hafour where the dominant habitats are woodland—*Quercus coccifera* and woodland—*Quercus infectoria* counting 10–11 genera in these sites (Figure 1a). At the opposite, the sites with the lowest CWR genus richness included Fahet Jernaya‐Beb El Hawa characterized with an oro‐Mediterranean shrublands, rocks, and screes habitat type, and the area of Marj El Tout where the dominant habitat is thickets of *Quercus* and *Crataegus* accompanied with agricultural lands. In these sites, the CWR genus richness did not exceed the range of 1–4 genera. Sites with CWR richness of 5–9 genera were found in the dominant habitats of: Woodland—*Quercus coccifera*, Woodland—*Quercus infectoria*, Woodland—*Prunus dulcis‐Quercus look*, and agricultural lands.

#### 4.3.2 CWR mapping based on species richness

Figure 1b shows that the 46 identified CWR species were mainly focused in Al Fakiaa and Sham El Hafour where the dominant habitats are respectively woodland—*Quercus coccifera* and agricultural land counting 23–28 species. Contrarily, areas with the lowest richness of 1–6 species were found in oro‐Mediterranean shrublands, rocks and screes, rocky grassland, woodland—*Quercus infectoria* and thickets of *Quercus* and *Crategus* habitat types. Ain Ata‐El Berke could be considered to have a moderately high CWR species richness counting between 18 and 22 CWR species in this site characterized as agricultural land.

#### 4.3.3 CWR mapping based on Shannon diversity index

The obtained values of Shannon diversity index varied from one habitat to another in the same cell as represented in the distribution map generated for representing CWR species diversity across the mountain habitats (Figure 1c).

### 4.4 Distribution of wild relatives of cereals

#### 4.4.1 Aegilops

**FIGURE 2:**
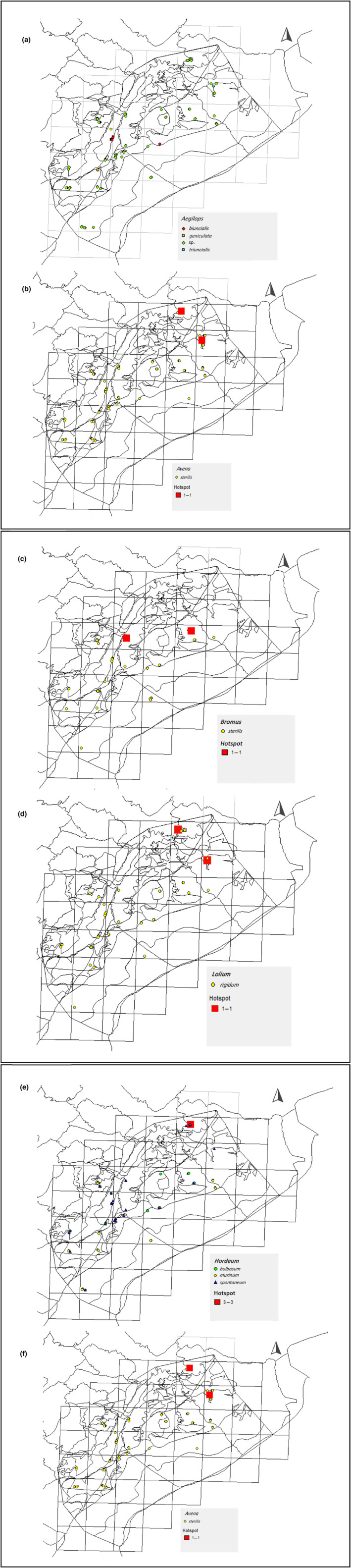
Distribution map of (a) *Aegilops* species, (b) *Avena sterillis*, (c) *Bromus sterillis*, (d) *Lolium rigidum*, (e) *Hordeum* species, and (f) *Hordeum* species richness.

*Aegilops* species occurred in all the 17 studied sites (Figure 2a) where *Ae. biuncialis* Vis, *Ae. geniculata* Roth., and *Ae. triuncialis* L. were found, while other occurrences were registered as *Ae*. sp. when certainty of species identification was not adequate.

#### 4.4.2 Avena

Only one species of *Avena* was found, *A. sterilis* L., with a wide distribution all over the mountain (Figure 2b) and mainly in the sites of Sahel Aiha‐Wadi el Byara and in Marj El Tout.

#### 4.4.3 Bromus

This genus was found under only one species, *B. sterillis* L. (Figure 2c). Although highly occurrent in sites that are identified as woodlands, Arid El Dalia and Rashaya El Wadi‐Wadi Sammouk.

#### 4.4.4 Hordeum

This genus is widely distributed throughout Mount Hermon and is found in all the habitats (Figure 2e). Three species were identified in different occurrence numbers simultaneously *H. murinum* subsp. *glaucum* (21), *H. bulbosum* L. (32), and *H. spontaneum* C. Koch (35) (Figure 2f). Species richness analysis shows that all three species of *Hordeum* are found in six sites, most importantly in Sahel Aiha‐ Wadi El Byara where all the species are found.

#### 4.4.5 Lolium

This genus occurred in all studied sites and habitats of the mountain, while only one species was identified, *Lolium rigidum* Goud. This species is mainly located in Sahel Aiha‐Wadi el Byara (agricultural land) and Marj El Tout (woodland) (Figure 2d).

#### 4.4.6 *Poa* and *Triticum*

Both genera occurred only one time during the survey, under the species *Poa pratensis* L. and *T. aestivum* L. therefore relevant information are too limited in this study.

### 4.5 Distribution of wild relatives of legumes

#### 4.5.1 Lathyrus

**FIGURE 3:**
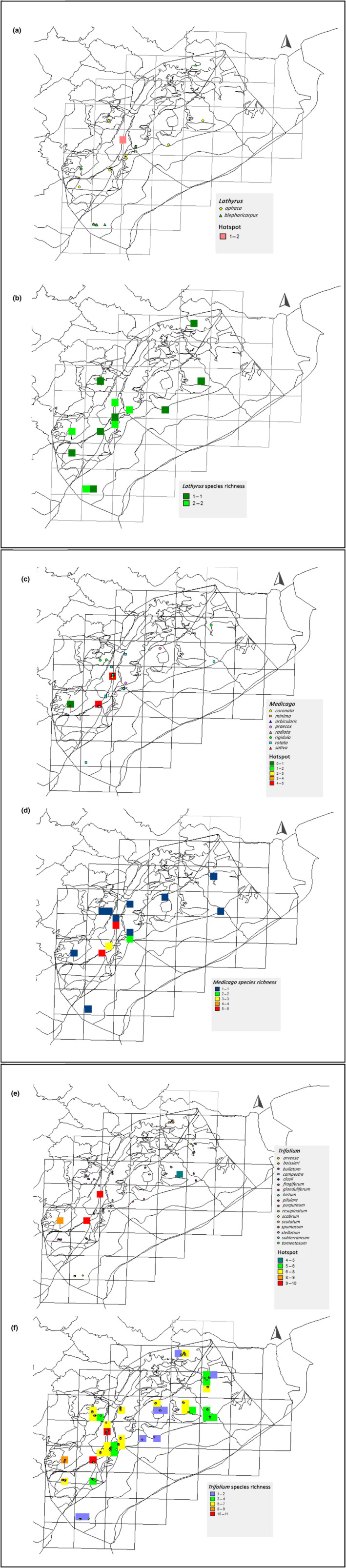
Distribution map of (a) *Lathyrus* species, (b) *Lathyrus* species richness, (c) *Medicago* species, (d) *Medicago* species richness, (e) *Trifolium* species, and (f) *Trifolium* species richness in Mount Hermon.

Distribution analysis for this genus shows its occurrence in 10 out of 17 surveyed sites (Figure 3a). *Lathyrus* species richness revealed two identified species, *Lathyrus blepharicarpus* Boiss. and *L. aphaca* L. (Figure 3b), that were both found at the same time in Al Fakiaa, Hima El Kadirin, Ain Ata el Berke characterized as woodlands, and also in Sham El Hafour and Chebaa‐Ouyoun Jenaa both considered as grassland and shrublands.

#### 4.5.2 Medicago

Species are distributed in a very random manner all over the different habitats of the Mount where 10 cells out of 17 reported this genus (Figure 3c). Eight different *Medicago* species were identified with *Medicago rotata* var. rotate Boiss. having the highest occurrence number, followed by *M. preacox* DC. with an occurrence value of 15, then *M. coronata* (L.) Bart., *M. orbicularis* L., and *M. rigidula* (L.) Desr, and finally with *M. radiata* L., *M. sativa* L., and *M. minima* (L.) Bart. displaying lowest occurrence values. Species distribution map shows that *M. sativa* L. was only registered in Ain Ata‐El Berke. Two major locations for high *Medicago* species richness were found in Al Fakiaa and Sham El Hafour sites with four to five species (Figure 3d).

#### 4.5.3 Trifolium

Species distribution map shows its occurrence in all surveyed areas except on the very high elevations of Oro‐Mediterranean shrubland, rocks, and screes habitat (Figure 3e). Seventeen species of *Trifolium* were identified. Four sites are designated as significant areas for these species, most importantly Al Fakiaa and Sham El Hafour where the following 14 species were found: *Trifolium boisseri* Guss., *T. bullatum* var. *bullatum*, *T. campestre* Schreb., *T. clusii* Gren. & Godr. var. *clusii*, *T. fragiferum* L., *T. hirtum* All., *T. pilulare* Boiss., *T. purpureum* Boiss., *T. resupinatum* var. *resupinatum*, *T. scabrum* L., *T. scutatum* Boiss., *T. stellatum* L., *T. subterraneum* var. *brachycalycinum* Katzn. & Morley, and *T. tomentosum* var. *glabrescens* (Post) Hausskn. & Bornm. Another major area is Ain Ata‐ El Berke counting nine species of *Trifolium*. Also, Rashaya Al Wadi‐Wadi Sammouk contained species not found in the previous two areas, such as *T. arvense* L. and *T. bullatum* Boiss. & Hausskn var. *bullatum*. Species richness maps also show sites where a number up to seven species of *Trifolium* was concentrated (Figure 3f).

#### 4.5.4 Vicia

**FIGURE 4:**
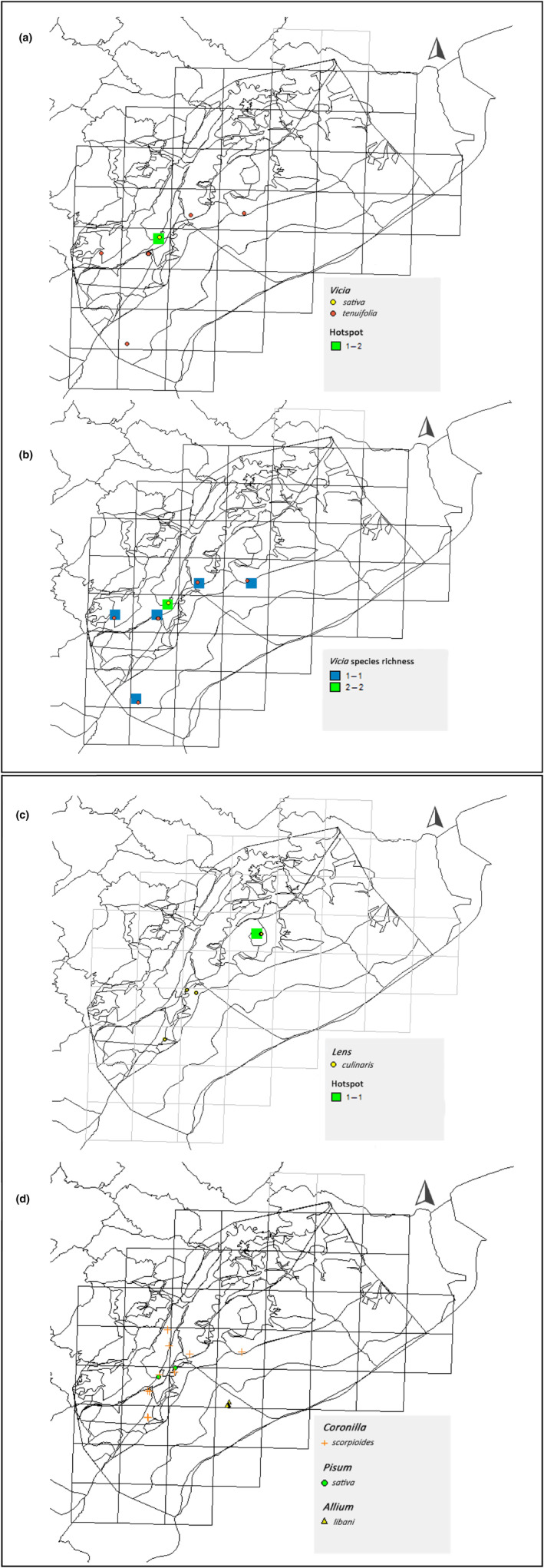
Distribution maps of (a) *Vicia* species, (b) *Vicia* species richness, (c) *Lens culinaris* subsp. *orientalis* (Boiss.) Ponert, and (d) *Coronilla scorpioides* L. and *Pisum fulvum* Sibth. & Sm in Mount Hermon.

*Vicia sativa* L. and *V. tenuifolia* Roth. were identified in a limited distribution mainly in woodlands of Sham El Hafour (Figure 4a). Richness map shows that both species are found at the same time in only one location (Figure 4b).

Data collected for *Lens*, *Coronilla*, and *Pisum* are limited. *Lens* (Figure 4c) was mainly found in woodland‐*Quercus infectoria* with only one species (*Lens culinaris* subsp. *orientalis* (Boiss.) Ponert), especially in Al Yebse‐Wadi El Botem (Figure 4d). *Coronilla scorpioides* L. also occurred primarily in woodland habitats, as well as *Pisum fulvum* Sibth. & Sm.

## 5 DISCUSSION

This study reports the first comprehensive distribution mapping of a group of cereal and legume CWRs in Mount Hermon (Rachaya side) with a particular focus on genus and species richness and diversity analyses being highly important in the protection and conservation of plant genetic resources for food and agriculture (Zair et al., 2020). The study highlights the importance of understanding CWR diversity and their distribution at both national and global levels. The species under study represent a substantial economic value to agriculture and agrobiodiversity noting that endangered food crop relatives have a worth of about USD 10 billion annually in wholesale farm values (Phillips & Meilleur, 1998).

CWR genus and species richness maps (Figure 1a, b; Table 3) show that considering areas of highest richness values, such as Sham El Hafour Al Fakiaa and Ain Ata‐al Berke, for a conservation plan could protect 40 out of the 46 targeted species. The remaining species either had low occurrences/frequency values in these two sites or their occurrence was prevalent in other study sites that are of specific habitat types. These findings are aligned with the study conducted by Bou Dagher Kharrat et al. (2018) that identified Mount Hermon as an important plant area due to its high richness index.

The analysis of sites with highest value of Shannon Index (Table 3) like Sham El Hafour (2.83) demonstrated the presence of 12 genera for 35 species, while each of Al Fakiaa (2.71), Ain Ata‐ al Berke (2.45), and Wadi El Raheb (2.41) sites retained nine genera designating, respectively 36, 24, and 21 species. Comparing the values of Shannon index found in Sham El Hafour and Al Fakiaa, where somewhat similar species richness values were found, higher densities of species were recorded in the former site. This result corroborates with findings of Akpo et al. (2000) that indicated a positive correlation between species richness and Shannon diversity index; the more numerous the species, the higher the Shannon‐Weaver index. Thus, some of the species, at least, may dominate whatever are the conditions, considering that the ecological systems having more species should be steadier, and if a disruption occurs in the environment, the dominant species will be able to protect the whole community. The detailed map generated for Shannon index distribution shows that the values vary within the same habitat in the same location (Figure 1c). This could be attributed to the extent of various threats, mainly overgrazing, in the areas surveyed on the Mount. Other reasons could be accredited to the ethnobotanical aspect of the mountain where some species are collected seasonally by the local due to their benefits (Baydoun & Arnold, 2016).

The high occurrence of *Aegilops* species in 106 out of 170 surveyed quadrates shows a significant dominance for these species in the Mount (Table 2; Figure 2a). These findings are in line with previous unpublished surveys (in ICARDA's database) of *Aegilops* species in Mount Hermon by ICARDA researchers in 2020 resulting in the identification of *Ae. biuncialis*., *Ae. cylandrica*, *Ae. ovata*, *Ae. Triuncialis*, and *Ae. columnaris* in areas that were not included in our survey. This clearly indicates the high prevalence of these species in Mount Hermon. It is essential to mention that during the period when the field survey was achieved, a prevalent extent of the grazing threat was observed, which could have affected the identification and finding of other *Aegilops* species. Lebanon is diverse in *Aegilops*, as other studies reported the occurrence of nine *Aegilops* species in Nabha, and up to six species in Arsal and Ham (Amri et al., 2005). Highlighting the occurrence of this genus is essential due to its close relationship with cultivated wheat which has brought this genus subject to numerous studies (Bolot et al., 2009; Kumar et al., 2019; Xie et al., 2010). It represents a source of vast genetic diversity and alleles of agronomic value to widen the wheat gene pool and improve tolerance to various types of potential stresses such pests, climate change, and other environmental stresses (Thiyagarajan et al., 2020). *Ae. biuncialis* Vis., specifically, is reported to be of a high potential for cross‐pollination with the cultivated wheat cultivated bread wheat (*T. aestivum*) in Italy in optimized experimentation for hybridization (experimental soil layout, flowering synchrony) (Loureiro et al., 2006). Among other priority CWRs, this species is red‐listed by IUCN meeting the category for Critically Endangered (CR) with high in situ priority (Perrino & Wagensommer, 2022). The flowering time of *Ae*. in Lebanon extends between April to June, depending on the species and its eco‐geographical zone; this would suggest a high in situ crossbreeding potential.

On the contrary to *Aegilops*, one occurrence only of *Triticum* species (*T. aestivum*) was registered (Table 2). These results could be explained by the sampling method as ICARDA has previously reported records of *T. urartu* and *T. dicoccoides* in the Mount including the herein study sites knowing that these species had been first reported in the Mount by Mouterde (1951). According to the literature, these species having different and interesting genomic constitutions that can induce resistance to various environmental stresses, are among the main gene pool of domesticated wheat (Pour‐Aboughadareh et al., 2021).

The presence of *A. sterilis* in all the habitats illustrated in Table 2 and Figure 2b aligns with Baum et al. (1972) stating the high adaptability of the genus considered a weed to a wide range of diverse habitats. A study of the phenotypic diversity of the species from Jordan revealed significant variations in different population traits illustrating the importance of considering various population clusters for gene enrichment in oat crop improvement (Al‐Hajaj et al., 2018).

Similarly, *Lolium rigidum* Goud. was abundant in all habitat types. Actually, this species is considered by Terrell (1968) as invasive with high adaptability to different conditions. Researchers have identified *L. rigidum* and *L. multiflorum* as the species evolving glyphosate resistance in California based on morphology and assumed history (Jasieniuk et al., 2008; Simarmata et al., 2003).

As for *Bromus*, one species (*B. sterilis* L.) was only identified in the surveyed sites of this study (Table 2; Figure 2c), while occurrences of other species such as *B. alopecuros*, *B. lanceolatus*, *B. tectorum*, and *B. tomentellus* were recorded in previous surveys conducted by Chalak et al. (n.d.) in similar habitats of the mountain.

With respect to *Lathyrus*, two species were identified in this study (Table 2; Figure 3a) as *L. aphaca* scored higher occurrence numbers and density than *L. blepharicarpus*. Currently 18 *Lathyrus* species are considered high priority species for conservation in the natural reserves of Lebanon like Horsh Ehden National Reserve and Arz Bcharreh National Protected Zone (Shehadeh et al., 2013). This does not affect the possibility that this species could be also conserved in Mount Hermon as climatic conditions vary widely between the two established reserves and our study area. Different adaptability traits are suggested for the same *Lathyrus* species (Aci et al., 2011). In preliminary studies of interspecific hybridization with *L. sativus* Sibth. & Sm. ex Steud. (grasspea), some wild *Lathyrus* L. have been found to be a potential germplasm resource in the breeding improvement programs of the grasspea (Kearney & Smartt, 1976; Yunus & Jackson, 1991).

In the case of *Medicago*, the species identified in our study had very different frequency and density values **(**Table 2 **)** as well as a random selectivity of distribution in habitat types (Figure 3c). According to Beyrouthy et al. (2019), 38 species of medics are inventoried in Lebanon, of which 20 were found in protected areas including three species identified in Rachaya. In our study, we were able to collect accurate georeferenced occurrence data for eight *Medicago* species of high priority that could be added to the Lebanese distribution map for medics.

The distribution of the 17 identified *Trifolium* species was found in all the habitats and surveyed altitudes except in Fahet Jernaya‐ Beb El Hawa (2254 m a.s.l.) (Table 2, Figure 3e). Similar distribution trends were obtained in the study conducted by Atallah et al. (2008) as most of the *Trifolium* species appeared in all surveyed altitudes in the south bank of Nahr‐Ibrahim valley. The absence of this species in the oro‐Mediterranean shrublands, rocks, and screes habitat type is aligned with the survey conducted by (Chalak et al., n.d.) in Mount Hermon where none of the *Trifolium* species were found in these habitats. The two areas previously identified in this study as retaining the highest CWR diversity, Sham El Hafour and Al Fakiaa, comprise 14 out of the 17 identified species of *Trifolium*.

The lowest occurrence records for *V. sativa*. and *V. tenuifolia*, *Lens culinaris* susbp. *orientalis*, *Coronilla scorpioides*, *Pisum fulvum*. shown in Table 2 and Figure 4a, c, d may be attributed to the fact that these legume species are among the favorite for pasture grazing.

One of the important achievements of this study was that we were able to survey a new site (i.e., Chebaa‐Ouyoun Jenaa) where no data was previously available. This site was the least explored region in the mountain due to security reasons. It lacked both historical and recent data (Bou Dagher Kharrat et al., 2018). In this site, we were able to identify 11 targeted species belonging to eight genera.

The Lebanese Parliament has voted to make Mount Hermon the country's 18th nature reserve. The designated protected area encompasses the sites we identified with highest species richness, Al Fakiaa, Sham El Hafour, and Ain Ata‐al Berke. To effectively manage CWR species within this reserve, threat, habitat, population, and genetic assessments must be conducted to inform the development of a targeted management plan (Iriondo et al., 2008).

In situ conservation can be achieved through two strategies. The first strategy involves the implementation of conservation actions in areas where high diversity, high genus, and species richness of CWRs have been detected. This strategy targets the sites of Al Fakiaa, Sham El Hafour, and Ain Ata‐al Berke. The second approach is at the genus level, a compilation of genus distribution maps and richness areas can be used to select conservation areas for projects focusing on specific genus or species. It is now an urgent priority to identify existing and novel mechanisms to finance and govern the global CWR network that will provide a fundamental basis for ensuring our future food security (Vincent et al., 2019).

## 6 CONCLUSIONS

Recognizing the crucial importance of the protection of plant genetic resources, this study reports the first comprehensive mapping of CWRs distribution in Mount Hermon (Bekaa side) with the aim of selecting areas having highest priority species richness and with the perspective of recommending monitoring and conservation actions.

These findings play a crucial role in mapping CWRs in Lebanon, which is essential for enhancing both in situ and ex situ conservation of CWRs and agrobiodiversity, both nationally and internationally.

Based on diversity analysis and richness maps, we have identified three sites in Mount Hermon (i.e. Sham El Hafour, Al Fakiaa, and Ain Ata‐ al Berke) to be recommended for active CWR conservation in situ. These sites cover species that are primarily adapted to habitats of Woodland—*Quercus infectoria* and Agricultural land and Woodland—*Quercus coccifera*. The implementation of a conservation strategy in these sites has the potential to conserve 40 species out of the 46 identified CWR species belonging to 13 genera in Mount Hermon. Therefore, it is recommended, as a part of a management plan, to establish national policies that cover these sites, thus allowing stakeholders to become aware of the existence of a national landmark with global genetic importance. For the 6 missing CWR species from the recommended conservation sites, it's suggested to implement collecting efforts in order to make sure they are also conserved in at least ex situ manner.

Richness distribution maps produced in our study provide a clear idea of sites with highest species density for each genus for further collection missions aiming at complementing the in situ with ex situ conservation actions. Ex situ conservation is crucial and a gap analysis is necessary to determine which species and populations are yet to be conserved in genebanks. This analysis should encompass the broadest range of each species' genetic diversity to ensure active conservation. If a species is not currently conserved ex situ, collection missions can be arranged using the maps produced in this study to target various habitats and genetic material for breeding programs. If it is already conserved ex situ, an evaluation of the collection date is required to determine if new collection missions are necessary. Additionally, collection sites should also be chosen based on different habitats to preserve a wide range of genetic diversity for breeding purposes.

## AUTHOR CONTRIBUTIONS

**Eliane Sayde:** Data curation (equal); formal analysis (equal); investigation (equal); project administration (equal); writing – original draft (equal). **Lamis Chalak:** Supervision (equal); writing – review and editing (equal). **Safaa Baydoun:** Project administration (supporting); resources (equal). **Ali Shehadeh:** Formal analysis (supporting); methodology (supporting); software (supporting). **Hicham El Zein:** Methodology (supporting); resources (supporting). **Jostelle Al Beyrouthy:** Project administration (supporting). **Mariana Yazbek:** Conceptualization (lead); funding acquisition (lead); methodology (lead); resources (lead); supervision (equal); writing – review and editing (equal).

## CONFLICT OF INTEREST STATEMENT

The authors have no conflicts of interest to declare.

## Supporting information


Appendix S1.

Appendix S2.

Appendix S3.

## Data Availability

The data that supports the findings of this study are available in the supplementary material of this article.
