# Supplementary material for: Surveying and mapping cereals and legumes wild relatives in Mount Hermon (Bekaa, Lebanon)
Source: Ecol Evol. 2024 Mar 11;14(3):e10943. doi: 10.1002/ece3.10943 (PMC10926055; doi:10.1002/ece3.10943)
Supplement: Supplementary file 2 — Appendix S2. [file ECE3-14-e10943-s002.docx]

**Surveying and mapping cereals and legumes wild relatives in Mount Hermon (Bekaa, Lebanon)**

**Supplementary figures**


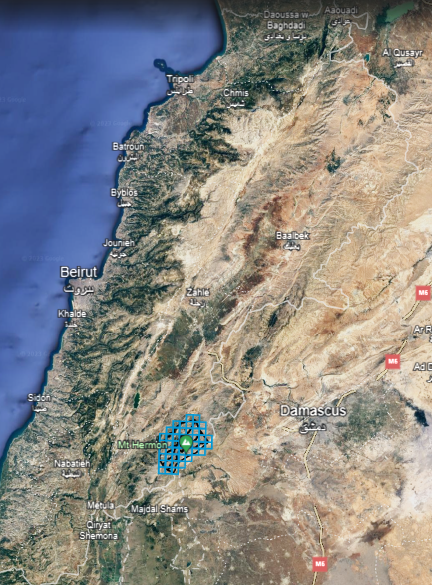


**Figure 1.** Map of Lebanon, with the study area highlighted with the respective grid in blue dividing the area into cells manageable for surveying.
