# Supplementary figures and images for: Surveying and mapping cereals and legumes wild relatives in Mount Hermon (Bekaa, Lebanon)
Source: Ecol Evol. 2024 Mar 11;14(3):e10943. doi: 10.1002/ece3.10943 (PMC10926055; doi:10.1002/ece3.10943)

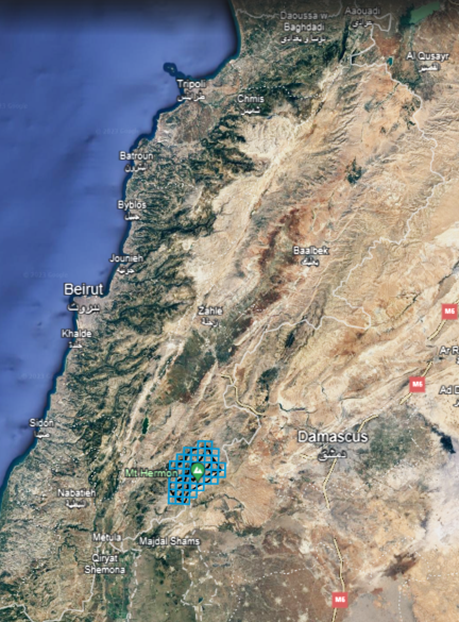

Supplement: Supplementary file 3 — Appendix S3. [file ECE3-14-e10943-s001.tif]
